# 非小细胞肺癌分子靶向治疗中EGFR-TKI的耐药机制研究进展

**DOI:** 10.3779/j.issn.1009-3419.2012.02.08

**Published:** 2012-02-20

**Affiliations:** 250117 济南，山东省肿瘤医院内科，山东省医学科学院 Department of Internal Medicine, Shandong Tumor Hospital, Shandong Academy of Medical Sciences Jinan 250117, China

**Keywords:** 表皮生长因子受体酪氨酸激酶抑制剂, 肺肿瘤, 耐药, 机制, Epidermal growth factor receptor-tyrosine kinase inhibitors, Lung neoplasms, Drug resistance, Mechanism

## Abstract

随着分子靶向治疗药物的发展，以吉非替尼（gefitinib, iressa）和厄洛替尼（erlotinib, tarceva）为代表的表皮生长因子受体酪氨酸激酶抑制剂（epidermal growth factor receptor-tyrosine kinases inhibitors, EGFR-TKIs）在治疗非小细胞肺癌中发挥了重要作用。然而在临床前和临床研究中发现许多患者对此药物存在原发性耐药或获得性耐药，使该类药物的使用受到一定限制。本文就近年来对EGFR-TKIs耐药机制的研究进展进行综述。

## Abstract (en)

With a greater understanding of tumor biology, novel molecular-targeted strategies that block cancer progression pathways have been evaluated as a new therapeutic approach for treating non-small cell lung cancer (NSCLC). Epidermal growth factor receptor-tyrosine kinase inhibitors (EGFR-TKIs), such as gefitinib and erlotinib, show favorable response to EGFR mutant lung cancer in some populations of NSCLC patients. However, the efficacy of EGFR-TKIs is limited by either primary (de novo) or acquired resistance after therapy. This review will focus on recently identified mechanisms of primary and acquired resistance to EGFR TKIs and strategies currently being employed to overcome resistance.

目前肺癌已成为全世界最常见的恶性肿瘤，其死亡率已居世界首位。肺癌中约80%-85%为非小细胞肺癌（non-small cell lung cancer, NSCLC），手术是治疗肺癌最有效的方法，但是大多数患者在确诊时已为晚期期而失去手术机会。以铂类为基础的化疗药物对NSCLC的治疗疗效虽有提高，然而这种疗法似乎进入了一个平台期，无法满足进展期NSCLC的治疗需要。近年来，肿瘤分子靶向治疗成为全球研究的热点之一，以吉非替尼（gefitinib, iressa）和厄洛替尼（erlotinib, tarceva）为代表的表皮生长因子受体酪氨酸激酶抑制剂（epidermal growth factor receptor-tyrosine kinase inhibitors, EGFR-TKIs）在NSCLC治疗中发挥了重要作用。但是在临床中，有些患者对EGFR-TKIs的治疗并不敏感，或开始对该类药物高度敏感但经过约10个月-14个月^[1, 2]^的中位无疾病进展生存期（progression free survival, PFS）后最终产生耐药，使该类药物的临床应用受到一定限制。阐明EGFR-TKIs的有效机制、原发性耐药机制和治疗有效后的继发性耐药（获得性耐药）机制，将有助于高选择性个体化治疗，为克服或逆转耐药提供新思路。本文将对近年来EGFR-TKIs耐药机制的研究进展作一综述。

## 1 EGFR结构及功能

EGFR是跨膜受体酪氨酸激酶家族的一员，该家族共4个成员，包括HER1（erbB1, EGFR）、HER2（erbB2）、HER3（erbB3）及HER4（erbB4），由3个不同的结构区组成，包括胞外配体结合区、跨膜区和胞内酪氨酸激酶区。EGFR可以与特异性配体如表皮生长因子（epidermal growth factor, EGF）、转化生长因子α（transforming growth factor α, TGF-α）、双向调节蛋白（AR）等结合形成同源二聚体，也可以和HER2或其它家族成员形成异源二聚体。二聚体的形成使蛋白质构象发生改变，导致酪氨酸激酶（tyrosine kinase, TK）激活及受体自动磷酸化，从而激活下游一系列信号转导通路，如Ras-Raf-MAPK途径、PI3K-Akt途径和STAT途径。EGFR信号通路在细胞的生长、增殖和分化等生理过程中发挥重要的作用，而在一些肿瘤细胞中EGFR常过表达，与肿瘤细胞的转移、侵润、预后差有关。

## 2 *EGFR*突变与EGFR-TKIs作用机制

肺癌中*EGFR*突变主要发生在胞内酪氨酸激酶编码区（18-21外显子），这些突变多存在于ATP结合裂隙附近，而该位点正是吉非替尼结合的靶位。其中最常见的有2种突变形式，占目前所有检测出来突变的80%-90%，分别为：①19外显子第746-750位密码子的缺失突变，导致EGFR蛋白中氨基酸序列丢失，改变了受体ATP结合囊角度；②21外显子点突变（L858R），此突变位点紧邻激酶活化环中高度保守的模体，两种突变均明显增强了肿瘤细胞对EGFR-TKIs的敏感性。临床研究显示：亚裔、女性、非吸烟、腺癌患者对EGFR-TKIs显示较好的疗效，且与*EGFR*突变分布一致。*EGFR*突变后对TKIs的反应率增高的可能解释有2种，一种是癌基因依赖学说，*EGFR*突变的肿瘤对EGFR这条信号通路高度依赖，所以阻断这条通路对肿瘤生长影响很大，从而提高了抗肿瘤药物的疗效。另一种可能是突变导致关键氨基酸残基重新分布，使得EGFR不管与ATP还是TKIs结合都更为紧密，这可以解释突变的EGFR与配体结合酪氨酸激酶活性增强，同时与TKIs结合后TK活性抑制也较强。

## 3 EGFR-TKIs耐药机制

### 3.1 原发性耐药

原发性耐药即肿瘤细胞在治疗初期就对TKIs治疗无明显反应，应用该药物治疗后病情未得到缓解，临床未明显获益。*EGFR*基因的突变率因种族的不同而不同，在亚洲NSCLC患者中高达20%-30%，而在高加索NSCLC患者中为10%-15%^[3]^。临床研究^[4]^证实，*EGFR*突变阳性的NSCLC对吉非替尼或厄洛替尼的治疗有效率为70%-80%，而野生型患者的有效率仅约10%-20%，因而，不少患者对EGFR-TKIs治疗存在着原发性耐药，而EGFR不同的基因状态可能存在着不同的原发性耐药机制。研究显示某些*EGFR*突变也可以引起NSCLC对EGFR-TKIs耐药，如治疗前存在EGFR 20外显子插入突变提示原发性耐药^[5]^，其机制可能是由于突变形成了空间位阻，使吉非替尼及厄洛替尼无法与TKs区结合，大大地降低了药物的敏感性。

但是上述突变较少见，只能对原发性耐药做出部分解释。*EGFR*突变的NSCLC对EGFR信号通路高度依赖，称为EGFR依赖或成瘾，当应用EGFR抑制剂时，这些通路被关闭而发生凋亡，然而*EGFR*野生型的NSCLC中没有此EGFR依赖来调控下游信号通路，可能伴有其它信号通路的异常，因此对EGFR-TKIs不敏感。研究^[6]^发现大约有10%-30%的NSCLC患者存在*KRAS*基因突变，这些突变与吸烟之间具有很强的相关性，而*EGFR*突变却常常发生在非吸烟者的亚裔患者中，这些数据说明*KRAS*和*EGFR*突变很少同时出现在同一类型的肿瘤中。另有研究^[7]^显示*KRAS*突变与*EGFR*突变互相排斥，也就是说该突变大多存在于*EGFR*野生型肿瘤，这可能是导致EGFR-TKIs原发性耐药的另一重要机制。*KRAS*基因突变主要发生于第12、13位密码子，这两种密码子主要发生G-T颠换，突变的结果导致编码的氨基酸发生改变，持续性激活EGFR非依赖性信号通路，导致瘤细胞增殖、转移以及凋亡抵抗，进而对EGFR-TKIs产生原发性耐药。*KRAS*突变在高加索人群NSCLC患者的突变率较高^[8]^，尤其是在肺腺癌患者中突变率达30%-50%，而*EGFR*的突变率较低（3%-8%），这可能是高加索人群对TKIs原发性耐药发生率较高。BR.21安慰剂对照试验^[9]^对*KRAS*与*EGFR*基因型对厄洛替尼治疗效果的影响进行了评估，结果显示，*KRAS*野生型患者（HR=0.69, *P*=0.03）可从厄洛替尼治疗中获得统计学意义上的生存益处，而*KRAS*突变型（HR=1.67, *P*=0.31）生存获益则未达到统计学意义。Sasaki等^[10]^分析研究了172例日本NSCLC患者的*KRAS*突变及其基因拷贝数的联系，以及与生存期的关系，结果显示仅*KRAS*突变与肺癌患者的生存期有关，*KRAS*突变及其基因拷贝数增加导致患者预后不良。因此，以上研究提示*KRAS*基因突变与肺癌患者对EGFR-TKIs的原发耐药有关，利用*KRAS*基因检测可以为TKIs药物治疗病人的筛选提供重要参考。

KRAS信号途径的其它信号分子可能与EGFR-TKIs的原发耐药有关，如*BRAF*（Raf亚型）突变。*BRAF*基因是RAF家族的成员之一，该家族还包括ARAF和RAF1。BRAF编码一种丝/苏氨酸特异性激酶，是RAS/RAF/MEK/ERK/MAPK通路重要的转导因子，参与调控细胞内多种生物学过程，如细胞增殖、分化和凋亡等，与肿瘤的发生关系密切。研究表明约3%的NSCLC存在*BRAF*突变，最常见的*BRAF*突变发生在BRAF激酶区活性部分的600位，由谷氨酸取代了缬氨酸（V600E），突变可激活MEK-ERK信号传导通路，诱导细胞增殖和分化。*BRAF*突变的NSCLC对EGFR信号通路缺乏依赖性，故对EGFR-TKIs有耐药性，而对MEK抑制剂敏感^[11]^。因此，应用靶向BRAF及整个RAF家族或它的效应器（如MEK）的药物，可对部分*BRAF*突变患者临床获益。

另外有研究^[12]^结果表明，棘皮动物微管相关蛋白样4（EML4）/间变淋巴瘤激酶（ALK）融合基因（E*ML4*-*ALK*融合基因）与TKIs耐药有关，约3%-5%的NSCLC患者可发现*EML4*-*ALK*基因重排，*EML4*-*ALK*基因重排常导致异常酪氨酸激酶表达。2009年Shaw等^[13]^分析了141例NSCLC患者*EML4*-*ALK*的突变情况，发现19例患者存在*EML4*-*ALK*突变（13%），这些多为不吸烟或轻度吸烟及腺癌患者，且EML-ALK阳性患者通常对TKIs治疗无效。因此需要转变对这部分患者的治疗策略，例如针对ALK的靶向治疗。

EGFR-TKIs原发性耐药也可能与非突变机制介导有关。在体外实验收集的资料^[14]^表明Met激活与EGFR-TKIs原发性耐药有关，与*MET*原癌基因扩增是相互独立的，实验还发现在原发肿瘤患者中Met的表达及磷酸化作用是发生脑转移的高危因素。与获得性耐药作用不同，原发耐药主要是由HGF激活MET通过GRB2相关结合蛋白1（GAB1）的作用激活下游信号通路^[15]^，而不是ErbB3的作用。

### 3.2 EGFR-TKIs的获得性耐药

获得性耐药即在治疗初期效果显著，经过几个疗程后对药物反应性下降，最终出现肿瘤复发或进展的情况。2010年Jackman等在*J Clin Oncol*杂志^[16]^上发表了关于EGFR-TKIs获得性耐药的定义：①既往接受过EGFR-TKIs单药治疗（吉非替尼或厄洛替尼）；②符合以下标准之一：具有与药物敏感性相关的EGFR基因突变（如G719X、19外显子缺失、L858R、L861Q）；接受EGFR-TKIs单药治疗后有客观上的临床获益，包括完全缓解、部分缓解或是超过6个月的疾病稳定（依照RECIST或WHO标准）；③接受吉非替尼或厄洛替尼连续治疗至少30天后出现疾病进展（RECIST或WHO标准）；④停用EGFR-TKIs及开始新的治疗方法之间无其它系统治疗。现有的研究显示，约50%的EGFR-TKIs获得性耐药与T790M突变相关，另有20%与原癌基因*MET*扩增相关，而一些获得性耐药的分子机制目前仍在研究中。

#### 3.2.1 T790M突变

EGFR二次突变是第一个被国际上认可的引起EGFR-TKIs获得性耐药的机制，率先由Kobayashi等^[17]^2005年发表在*N Engl J Med*上的文章提出。该研究对1例治疗前有EGFR 19外显子敏感活化突变（del L747-S752）、接受吉非替尼二线治疗达完全缓解24个月后出现进展的肺腺癌患者进行二次活检，分析其活检组织*EGFR*基因突变情况，发现在原有突变基础上，*EGFR*基因发生了第二次突变，突变位点在20外显子，即酪氨酸激酶活化域的790位苏氨酸残基被甲硫氨酸取代，从而形成位阻效应，在空间上阻碍了吉非替尼和厄洛替尼的连接。近年来的研究^[18]^显示，T790M突变增加了EGFR与ATP之间的亲和力，相对削弱了与EGFR-TKIs的亲和力。因此针对T790M突变导致TKIs耐药的NSCLC，可给予不可逆性的多靶点抑制剂作为后续治疗。

T790M突变是怎样产生的呢？为了说明这一问题，2006年Inukai等^[19]^用一种使T790M突变富集的高敏感性PCR检测方法，从280例未经吉非替尼治疗的NSCLC患者中发现9例T790M突变（3.6%），而用直接测序方法仅检出1例，据此作者提出了假说：经过EGFR-TKls治疗后敏感肿瘤细胞被杀灭，而其有T790M突变的耐药肿瘤细胞则被选择性存活下来。Maheswaran等^[20]^通过用一种高敏感的PCR方法，对26例具有*EGFR*突变的NSCLC患者外周血循环肿瘤细胞（circulating tumor cell, CTC）及肿瘤组织进行了分析，有10例在血液中检测出了具有T790M突变的肿瘤细胞（38%），且只有以很高的扩增循环数才能检测到，发现该突变不仅存在于接受EGFR-TKIs治疗的患者肿瘤细胞中，还存在于治疗前的肿瘤组织中，突变表明PFS持续时间更短。以上研究结果表明，T790M突变在未经EGFR-TKIs治疗的肿瘤细胞中有微克隆存在，治疗过程中这些克隆被选择性富集。

#### 3.2.2 MET扩增

原癌基因*MET*扩增是导致EGFR-TKIs获得性耐药性的另一重要机制。MET是肝细胞生长因子（HGF）/分散因子的一种高亲和力的酪氨酸激酶受体，被激活后发生自身磷酸化，引起多种底物蛋白磷化和细胞内一系列信号传导，激活MAPK-ERK1/2及P13K-Akt等信号通路，促进多种瘤细胞的生长、侵袭和转移。2007年Engelman等^[21]^构建了一株开始对吉非替尼敏感的但暴露在递增浓度的吉非替尼6个月后产生对其耐药的克隆的细胞株，结果发现这一耐药归因于*MET*原癌基因的扩增。研究者提出MET扩增导致的吉非替尼耐药是通过以ErbB3介导的PI3K通路持续激活，从而绕过了受抑制的EGFR靶点而产生。

由于MET扩增可促进TKIs耐药性的产生，多个MET抑制剂正在研究中，如HGF抗体（AMG102）、MET抗体（MetMAb）及MET小分子抑制剂（XL184、ARQ 197、SGX-523等）。其中ARQ197是一种选择性的MET-TKIs，应用于晚期NSCLC的Ⅱ期临床研究^[22]^显示，厄洛替尼+ARQ197组的PFS为16.1周，而厄洛替尼+安慰剂组为9.7周。联合EGFR-TKIs和c-MET抑制剂可能延缓耐药，但有待进一步研究结果证实。

#### 3.2.3 其它机制

对TKIs获得性耐药的患者，约30%-40%既无继发突变，也无MET扩增，这些患者耐药机制的研究还在进行中。可能的机制如下：

肿瘤细胞从上皮细胞向间充质细胞转化（epithelial-mesenchymal transition, EMT)，是生物胚胎形成和发育常见的生理过程，同时还存在于多种病理过程中，与肿瘤细胞的原位侵袭和远处转移有着密切的关系。EMT以上皮标记物蛋白（如E-钙粘蛋白、EpCAM等）的下调及间叶标记物蛋白（如波形蛋白、纤连蛋白等）的上调为特征。研究^[23]^发现，当发生EMT的肺癌重新表达E-钙粘蛋白后，肿瘤细胞可恢复对EGFR-TKIs治疗的敏感性。Yauch等^[24]^收集了87例化疗联合厄洛替尼治疗后复发的肺癌患者，对其中的65例进行分析后发现，E-钙粘蛋白高表达的患者PFS更长（HR=0.37），而对厄洛替尼不敏感的肺癌组织往往合并波形蛋白和（或）纤连蛋白等标记物的表达。以上研究提示EMT状态可作为预测EGFR-TKIs疗效的潜在因素，这仍需进一步研究确定是否有临床意义^[25, 26]^。

胰岛素样生长因子1受体（insulin-like growth factor 1 receptor, IGF-1R）是一种跨膜蛋白，在致癌性转化、肿瘤细胞生长和存活起到非常重要的作用。有研究^[27]^指出，EGFR-TKIs可诱导NSCLC细胞膜表面EGFR/IGF1R异源二聚体化进而激活IGF-1R及其下游信号通路MAPK和P13K/AKT的转导，并能刺激mTOP介导的survivin蛋白合成引发抗凋亡效应，从而引起肺癌患者对TKIs耐药。另一项体外研究^[28]^显示，对吉非替尼耐药的细胞株中，IGF结合蛋白3（IGFBP3）及IGF结合蛋白4（IGFBP4）表达下调，与IGF-1R及其下游信号通路转导的激活有关。EGFR高表达的样本中也伴随着IGFR-1的高表达，因此EGFR和IGF-1R双靶点抑制剂可作为潜在的治疗策略。

另外EGFR-TKIs获得性耐药也与以下几个方面有关：①研究发现*PTEN*基因表达丧失可活化PI3K/Akt/mTOR信号通路，导致凋亡抵抗，促进癌症的发生，进而对EGFR-TKIs产生耐药^[29-31]^；②有报道^[32]^肿瘤细胞微环境改变与获得性耐药有关，可能与TGF-β及IL-6的分泌有关；③EGFR-TKIs可诱导FGFR2和FGFR3信号通路，从而引发对其产生快速的获得性耐药^[33]^，联合应用EGFR和FGER酪氨酸酶抑制剂，可逆转TKIs耐药；④近来有研究报道^[34]^，*EGFR*野生型患者中BCRP/ABCG2高表达可对吉非替尼产生获得性耐药。细胞信号传导过程复杂，具有多向性，因此对EGFR-TKIs耐药机制的研究仍是当前靶向治疗领域的探索热点。

## 4 展望

EGFR-TKIs虽然在晚期NSCLC治疗中发挥了重要作用，但其原发与获得性耐药问题已经成为限制其疗效进一步提高的瓶颈，因此对其耐药机制的深入研究，寻找克服耐药的治疗方法，已经成为肿瘤研究领域的迫切任务。随着基础研究和临床实验的不断深入，EGFR-TKIs药物耐药机制逐渐清晰，越来越多的针对肿瘤耐药机制或作用于其它相关信号通路的靶向药物逐渐进入临床，并取得一定疗效。

目前针对EGFR-TKIs耐药问题，临床治疗策略主要涉及以下方面：①研究发现不可逆的EGFR-TKIs可用于对抗有*EGFR*突变的NSCLC患者的吉非替尼和厄洛替尼的耐药性。与可逆性TKIs不同的是，不可逆的EGFR-TKIs与EGFR TK区永久性结合；②“旁路激活途径”在EGFR-TKIs耐药中发挥重要作用，肿瘤细胞信号传导相互交错，单靶点药物不能阻断肿瘤细胞所有信号转导，因此开发多靶点的靶向治疗药物成为新的研究趋势^[35]^；③某些生物学分子标记物与EGFR-TKIs疗效相关。为使EGFR-TKIs的临床应用更为合理有效，需确定有效的预测靶标及最佳的检测方法，选择适合的患者接受TKIs治疗，进一步提高NSCLC的疗效和生存，最大限度地避免无效的治疗。随着对EGFR-TKIs原发性和获得性耐药机制的不断深入探讨，与EGFR-TKIs疗效有关的分子生物标记及其之间的相互关系必将越来越明了，有望使个体化治疗更上一个台阶。
